# Supplementary material for: Peptidoglycan recognition protein PGRP-5 is involved in immune defence and neuro-behavioral disorders in zebrafish embryos
Source: PLoS One. 2025 Jan 31;20(1):e0315714. doi: 10.1371/journal.pone.0315714 (PMC11785313; doi:10.1371/journal.pone.0315714)
Supplement: S1 File — (DOCX) [file pone.0315714.s001.docx]

>Dr_PGRP2

MAGICMQSSAVHLRNMEHFITAVAHLEHLNPQLSSLALVSNLCQIADQTHNVTHGFMRSSNNETETHPKIKERLNINLNLSHFFSQALHHFITAAYEEKGVVLTPDGTTVALAPLLLGLESGLRAENVSSQPNGFYLHPLARTLGLSFLNSTPTVRLGAEGCWDSVSAPMVFTLSNFPSLVTDAVVHGGMDGAILGKHLSVVNCSKMNVSTLLRSYYLRTVEELDDVLDPHLKGRNRRQNFQKITSSSLLQEKVAGVLPSQGDAELESLIAKGIKEFVLRYMDCPSIIPRCIWGAAPPQVPLELLSPPMSFLYIHHTAIPSKPCLNLQTCSQNMRAMQRFHQKDWGWYDIGYSFVVGSDGYIYEGRGWMSQGAHTKGRNNVGYGVAFIGDYSGRLPSTHDMELVRHHLVKCGVNNGFLQEDFTILGHRQVVVTTSCPGNALYSEITTWMHYKDKDPLK

>Dr_PGRP5

MQHSFFIFLYTGAEFLHETGKYAEVHVCCPSLSSRANKPVSLVMLEHTGNEKFVADMDGHTNTVDINADTVSRRGWDAVQPREMTQMESPAHTVIVHHTALRFCAHPRESVTELAHIQRMHMQERGFDDIGYNFLISGDGTVYEGRGWGIVGAHAKEHNFYSVGIAFMGNLNADLPSSASLSALLRLLHIGVLHGHVRPNFVLLGHKDVAKTACPGENLYSVLPKLRDRLQNNELLQA

>Dr_PGRP6

MMGRQFISVCLRKGQDGFKRRASSEANTQSDFIIRGYLALNYSVFMMDLRLFSLLMLFACGIAEATTLKRMDDFISAVESIEAVNPALSMLDVLKGLRKASGLETDLSQQLLGDAHPELTMDPSVASNIHELINHHVSDITEEGVVLTLDGSNVALAPLLLGLEAGLQSIDLYPLTLTENLLNSFVHHVQNQESSALLGTKGYWDSIKSPRVYTLSGLPSLATDALIIGGMDGFILGSEMASSDHPEESLSSLLRSYYSQKLDDAAPRLISQKRRMNFRNMADFSLMKTQVVRALTVRRNLNQDERKKLDDVVNEGFEEFVHVYAVCPNIITRSQWGAASYIGSPSYLSLPVRYLFIHHTYQPSKPCTTFEQCAAEMRSMQRYHQQSNGWSDIGYSFVAGSDGNLYEGRGWNWVGAHTYGYNSIGYGVCFIGDYTSTLPASSAMNMVRYDFTYCATNGGRLSKSYSLYGHRQAAATECPGNTLYRQIQTWERYQSYLP

>Hs_PGRP1

MSRRSMLLAWALPSLLRLGAAQETEDPACCSPIVPRNEWKALASECAQHLSLPLRYVVVSHTAGSSCNTPASCQQQARNVQHYHMKTLGWCDVGYNFLIGEDGLVYEGRGWNFTGAHSGHLWNPMSIGISFMGNYMDRVPTQAIRAAQGLLACGVAQGALRSNYVLKGHRDVQRTLSPGNQLYHLIQNWPHYRSP

>Hs_PGRP2

MAQGVLWILLGLLLWSDPGTASLPLLMDSVIQALAELEQKVPAAKTRHTASAWLMSAPNSGPHNRLYHFLLGAWSLNATELDPCPLSPELLGLTKEVARHDVREGKEYGVVLAPDGSTVAVEPLLAGLEAGLQGRRVINLPLDSMAAPWETGDTFPDVVAIAPDVRATSSPGLRDGSPDVTTADIGANTPDATKGCPDVQASLPDAKAKSPPTMVDSLLAVTLAGNLGLTFLRGSQTQSHPDLGTEGCWDQLSAPRTFTLLDPKASLLTMAFLNGALDGVILGDYLSRTPEPRPSLSHLLSQYYGAGVARDPGFRSNFRRQNGAALTSASILAQQVWGTLVLLQRLEPVHLQLQCMSQEQLAQVAANATKEFTEAFLGCPAIHPRCRWGAAPYRGRPKLLQLPLGFLYVHHTYVPAPPCTDFTRCAANMRSMQRYHQDTQGWGDIGYSFVVGSDGYVYEGRGWHWVGAHTLGHNSRGFGVAIVGNYTAALPTEAALRTVRDTLPSCAVRAGLLRPDYALLGHRQLVRTDCPGDALFDLLRTWPHFTATVKPRPARSVSKR

SRREPPPRTLPATDLQ

>Hs_PGRP3

MGTLPWLLAFFILGLQAWDTPTIVSRKEWGARPLACRALLTLPVAYIITDQLPGMQCQQQSVCSQMLRGLQSHSVYTIGWCDVAYNFLVGDDGRVYEGVGWNIQGLHTQGYNNISLGIAFFGNKIGSSPSPAALSAAEGLISYAIQKGHLSPRYIQPLLLKEETCLDPQHPVMPRKVCPNIIKRSAWEARETHCPKMNLPAKYVIIIHTAGTSCTVSTDCQTVVRNIQSFHMDTRNFCDIGYHFLVGQDGGVYEGVGWHIQGSHTYGFNDIALGIAFIGYFVEKPPNAAALEAAQDLIQCAVVEGYLTPNYLLMGHSDVVNILSPGQALYNIISTWPHFKH

>Hs_PGRP4

MLPWLLVFSALGIQAWGDSSWNKTQAKQVSEGLQYLFENISQLTEKGLPTDVSTTVSRKAWGAEAVGCSIQLTTPVNVLVIHHVPGLECHDQTVCSQRLRELQAHHVHNNSGCDVAYNFLVGDDGRVYEGVGWNIQGVHTQGYNNISLGFAFFGTKKGHSPSPAALSAMENLITYAVQKGHLSSSYVQPLLGKGENCLAPRQKTSLKKACPGVVPRSVWGARETHCPRMTLPAKYGIIIHTAGRTCNISDECRLLVRDIQSFYIDRLKSCDIGYNFLVGQDGAIYEGVGWNVQGSSTPGYDDIALGITFMGTFTGIPPNAAALEAAQDLIQCAMVKGYLTPNYLLVGHSDVARTLSPGQALYNIISTWPHFKH

>Mm_PGRP1

MLFACALLALLGLATSCSFIVPRSEWRALPSECSSRLGHPVRYVVISHTAGSFCNSPDSCEQQARNVQHYHKNELGWCDVAYNFLIGEDGHVYEGRGWNIKGDHTGPIWNPMSIGITFMGNFMDRVPAKRALRAALNLLECGVSRGFLRSNYEVKGHRDVQSTLSPGDQLYQVIQSWEHYRE

>Mm_PGRP2

MKAWGALWIVLGLLLWPEPGAASSLPLLMDSIIQALAELEQKVPVTEASITASAWILSAKNSSTHNSLHQRLLLKAPSHNTTEPDPHSLSPELQALISEVAQHDVQNGREYGVVLAPDGSTVAVKPLLFGLEAGLQAHSVANLPSDCLAIPCDTGDTLANIRATWPGLMDAFPNASSPDVGATLPNDKAKTPTTVDRLLAITLAGDLGLTFLHRSQTWSPPGLGTEGCWDQLTAPRVFTLLDPQASRLTMAFLNGALDGALLGNHLSQIPRPHPPLSHLLREYYGAGVNGDPVFRSNFRRQNGAALTSAPTLAQQVWEALVLLQKLEPEHLQLQNISQEQLAQVATLATKEFTEAFLGCPAIHPRCRWGAAPYRGHPTPLRLPLGFLYVHHTYVPAPPCTTFQSCAADMRSMQRFHQDVRKWDDIGYSFVVGSDGYLYQGRGWHWVGAHTRGYNSRGFGVAFVGNYTGSLPNEAALNTVRDALPSCAIRAGLLRPDYKLLGHRQLVLTHCPGNALFNLLRTWPHFTEVEN

>Mm_PGRP3

MLVSWDHPKMLPRLLGFLALSLLACGNPTIVSRKEWGASSLTCRVPLSLPVPYLIIEQVTRMQCQDQITCSQVVRVLQSQYVHNKGWCDIAFNFLVGDDGKVYEGVGWYVQGLHTQGYNNVSLGIAFFGSKIGSPSPAALSATEDLIFFAIQNGYLSPKYIQPFLLKEETCLVPQHSEIPKKACPNITPRSAWEARETHCPQMNLPAKFVIIIHTAGKSCNESADCLVRVRDTQSFHIDNQDFCDIAYHFLVGQDGEVYEGVGWNIEGSHTYGYNDIALGIAFMGNFVEKPPNEASLKAAQSLIQCAVAKGYLTSNYLLMGHSDVSNILSPGQALYNIIKTWPHFKH

>Mm_PGRP4

MLSWLLVFSILVLLAQGVSSWENPQTDQVSEGLQQLFGNISQLFEKGILGRDDVFTMVSREEWGAEAIGCSSKLSRPVDVLVIHHIPGLECHNKTVCSQKLRELQAYHIHNSWCDVAYNFLVGDDGRVYEGVGWNVQGSHDQGYKNISLGVAFFGTQEGHSPSPVALSAMKGLISYAVKKGHLSSKYIQPLLAKSEDCLVPPQKGKQKKACPHIVPRSVWGARDSHCSRMTLPAKYAIILHTAGRTCSQPDECRLLVRDLQSFFMNRLNACDIGYNFLVGQDGGVYEGVGWNNQGSKTDSYNDISLSITFMGTFTGSPPNAAALEAAQDLIRCAVVKGYLTPNYLLMGHSDVSNTLSPGQALYNIIKTWPHFKH

>Ol_PGRP2

MTPFVPACFVVILFHFSAVCSRPTGAHLRNMESFIEAVREVEDSTPGLSPLDLLRALRKTAGHDDVMTTHFLGESYNFSGSDVLEKAILNASSFSFFDKAIHHIVTDRGEERGVVLAPDGTTVALAPLLLGIELGLKAKVEGGSPVGIFPITLGRRLGLTFLSLQDFPASVRLGPNGCWDDVERPKVYKLSRHATLATDAIINGGMDGLILGMDLNSPAAPEQTRALSEILKGFYFFTLQEQGLDAVATHISPKRRELSKSLLESSDLHITVMETLALVWKLEKTEWIALDTGVGKAVKDGLQEFVHKYWDCPQIIPRCQWGAKPQQGTPVPLSLPLQYLYIHHTYLPSSPCLSFPNCSRDMRSMQRFHQEDRGWDDIGYSFVVGSDGYIYEGRGWTYRGTHTRGHNSIGYGVAVIGNYTASLPSRHATRLLRDRLAQCAVDGGALAANFSIQGHRQVVNYTTCPGDAFFGEIKTWEHFRE

>Ol_PGRP5

MEQTVNVVSRLQWGAAAPKQKEALKGSAQRVVIHHTALQNCRGLADSKEHLVSIQSMHMNQRGFDDIGYN

FLVGGDGTVFEGRGWGVVGAHTKGHNHDSLGIAFMGNFNSDKLSKEALLSVKRLLLFGVSQSYIHPKFVLCGHRDLASTQCPGDNLYAALTHLRSAA

>Ol_PGRP6

MKEGGWKLTLAVLVLLVSTHADALFSCHMKDFIKAVQQVEDENPELEAVEVLRMLRRTAHLNDAFVQHFLPDAGSSGPELDADLSGYLQRAVQHRVLEGAREEGVVLAPDGMTVAVGPLLLGIEAGFLSTIPGRVPGLYQLTLAGDLGVSIRRRSENGEPLGIDGCWDNLTAPQVFTLSDTASTLTAAQVHGGMDGVILGKEVSAKAGESVKLSSLLTDYYLHQLDAKGMDGAPRLISRRRRELFKILVAPPVLIRQVAKSMELREKLEGRVKMEVRQKRRLMAAVKETMKEFVHVFMECPPIIARCTWGAAPYIGTPTMLSLPLTYLFIHHTASPSQPCLTFEQCSADMRSMQRFHQQTNGWDDIGYSFVAGSDGNIYEGRGWKWQGAHTGGYNSKGYGVSFIGDYTSTLPSQHAMALVRDQLASCAVAGGQLVSSYILKGHRQMVSTECPGNIFYKEITTWEHYQP
