# Supplementary figures and images for: Peptidoglycan recognition protein PGRP-5 is involved in immune defence and neuro-behavioral disorders in zebrafish embryos
Source: PLoS One. 2025 Jan 31;20(1):e0315714. doi: 10.1371/journal.pone.0315714 (PMC11785313; doi:10.1371/journal.pone.0315714)

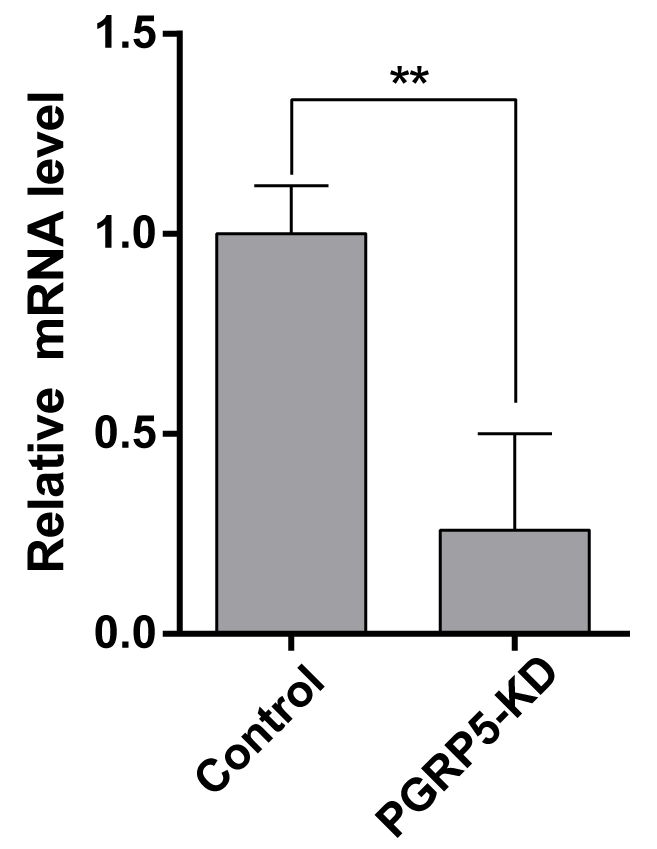

Supplement: S1 Fig — (TIF) [file pone.0315714.s002.tif]
