# Supplementary material for: Peptidoglycan recognition protein PGRP-5 is involved in immune defence and neuro-behavioral disorders in zebrafish embryos
Source: PLoS One. 2025 Jan 31;20(1):e0315714. doi: 10.1371/journal.pone.0315714 (PMC11785313; doi:10.1371/journal.pone.0315714)
Supplement: Attachment — Submitted filename: Reviewers comment_PONE-D-23-37049_31 May 24 for Author.docx [file pone.0315714.s003.docx]

**Manuscript Number:** PONE-D-23-37049

**Full Title:** Peptidoglycan recognition protein PGRP-5 is involved in immune defense and neurobehavioral disorders in zebrafish embryos

**Reviewer’s comments**

A. **Title** (Line 1-2)
1. Appropriately written. Replace s with c in defence
B. **Abstract –**
1. Concise and reflect the content of the works done.
2. Line (27-28) – Cytokines are italicised here. It should be followed in the entire manuscript.
C. **Keywords**
1. Selection of keywords reflect the content completely.
D. **Introduction**
1. Line 50-51- Write immune response *against* instead of *of* .
2. Line 51 – Write full form of IMD
3. Line 63 - *and structure also indicates.* Add its before structure
4. Line 66 - challenge *of* pathogenic. Write due to instead of *of.* Add further before *The*
5. Line-75 - I should be small in intracellular
6. Line-81 – development and behaviour, *of whom?*
7. Line-83 – microbiota-brain interactions, *of whom?*
8. Line-85 – development and behaviour, *of whom?*
9. Line-85-87 – In…sentence is not complete.
10. Line 93- remove *further* after we
11. Line-96- replace *aquatic ecosystem* with fish
E. **Materials and Methods**
1. Line-142 – Write complete name of E. coli and M. lutues and *italicize* it.
2. Line-143 - 1 x 107 CFU/mL. it should be superscripted.
3. Line-162 - -actin…??
4. Line-168&181 – 500 L…??
F. **Results**
1. Line – 233 – write potential instead of potentially
2. Line-241- write Gram - before positive
3. Line-257 – TNF- α
4. Line-281- locomotor not loco-motor

DEAR AUTHOR, YOUR MANUSCRIPT AND FINDINGS ARE WRITTEN CORRECTLY. PLEASE DO FEW CHANGES SUGGESTED TO YOU.

- For editor

Dear Editor, I have gone through paper (Title, abstract, Keywords, Introduction, M&M, results and discussion). I found that the finding is new and interesting. There are few typographical error committed by the authors and I have suggested to correct it. Further, the authors have written the manuscript in Standard English, followed referencing guidelines and step by step presentation of the write up. Hence, I recommend to accept the manuscript.
