# Supplementary material for: Peptidoglycan recognition protein PGRP-5 is involved in immune defence and neuro-behavioral disorders in zebrafish embryos
Source: PLoS One. 2025 Jan 31;20(1):e0315714. doi: 10.1371/journal.pone.0315714 (PMC11785313; doi:10.1371/journal.pone.0315714)
Supplement: Attachment — Submitted filename: Response letter.docx [file pone.0315714.s005.docx]

**Response Letter**

Dear editor and reviewers,

We are happy to send this revised version R3 of the manuscript entitled “**Peptidoglycan recognition protein PGRP-5 is involved in immune defence and neuro-behavioral disorders in zebrafish embryos**”. We appreciate that our study has been found to be of interest, bringing novelty of relevance to the area of neurodevelopment and immune inflammation in aquatic organisms.

We take the opportunity to thank the editor and the reviewers for their time and expert appraisal of our study. We believe that the points raised by the reviewers have been adequately addressed in this revised version, as seen by the changes detailed and highlighted by the red color text in the R3 manuscript.

The detailed point-by-point responses are listed below.

**Additional Editor Comments:**
The manuscript has been revised accordingly. The authors have addressed all the comments from the reviewers. However, there are some details that the authors should pay attention to. Therefore, I invite the authors to revise the details before the manuscript is accepted.
1. Beside the biological replicates, the technical replicates should be mentioned in M&M and the captions, such as Fig. 3, Fig. 4, Fig. 5, Fig. 6.
**Answer:** Thank you for your suggestions. Each value contains at least four biological replicates, and each biological replicate contains three technical replicates. We have added these information in the Fig. 3, Fig. 4, Fig. 5, Fig. 6 and M&M sections.
2. In Fig. 3A, what does the green and pink boxes stand for? Please indicates in the text and captions. Besides, the PGRP domain should be boxed as well.

**Answer:** Thank you for your suggestions. The green boxes represent the low complexity region, and pink boxes stand for PGRP domains that contained a conserved N-acetylmuramoyl-L-alanine amidase (ami_2) region. We have added these information in the legend of Fig. 3A.
3. Please explain and discuss why AChE was up-regulated in PGRP5-KD?

**Answer:** Thank you for your suggestions. The up-regulation of Acetylcholinesterase (AChE) in PGRP5 knockdown (KD) could potentially be attributed to several factors. One possibility is that the knockdown of PGRP5 might disrupt normal cellular signaling pathways that regulate the expression of AChE. PGRP5 could be involved in a complex network of interactions and its knockdown could lead to compensatory mechanisms or imbalances in downstream signaling, resulting in the increased expression of AChE. Another consideration is that PGRP5 might directly or indirectly interact with transcription factors or regulatory elements that control the expression of the AChE gene. The absence or reduced function of PGRP5 could remove an inhibitory effect or trigger a feedback loop that promotes AChE up-regulation.

**Reviewer #1:** I appreciate the diligence of the authors to address my points in the previous submission, and now feel these works are ready for publication. I do have a few minor comments below:
Minor:
- Fig3 B the intestine x-axis line is not straight

**Answer:** Thank you for your reminding. We have revised the intestine x-axis in Fig. 3B.
- Line 146, suggest to revise “in a nutshell…” vernacular to something like “Briefly,”

**Answer:** Thank you for your reminding. We have made the corresponding revisions according to your suggestions.
- Fig4 B y-axis label revise – states “Percent \ %”

**Answer:** Thank you for your reminding. We have made the corresponding revisions according to your suggestions.
- It would be interesting to challenge PGRP-5 KD fish vs scrambled with E coli or M luteus as a definitive demonstration that loss of PGRP-5 leads to even more killing and reduced rate of hatching in fig4

**Answer:** Thank you for your suggestions. What we want to demonstrate in Fig. 4 is that the Dr-PGRP5 gene exhibits different expression patterns under the stimulation of Gram-positive and Gram-negative bacteria, respectively. Meanwhile, *M.luteus* bacteria had lower survival and hatching rates compared to *E. coli* bacteria on normally developing zebrafish embryos. Actually, we did not test whether PGRP5-KD fish resulted in lower survival and hatching rates compared to normal fish, which is indeed an interesting question and we will explore this issue in future studies.
- In 3.3 and throughout the manuscript be consistent with stating “Gram positive” / “Gram negative” or “Gram-positive” / “Gram-negative”

**Answer:** Thank you for your suggestions. “Gram-positive” / “Gram-negative” were consistent throughout the entire manuscript.
